# Is arbuscular mycorrhizal fungal addition beneficial to potato systems? A meta-analysis

**DOI:** 10.1007/s00572-024-01178-0

**Published:** 2024-12-16

**Authors:** Segun Oladele, Iain Gould, Sandra Varga

**Affiliations:** 1 School of Natural Sciences, University of Lincoln, Lincoln, LN6 7TS UK; 2 Lincoln Institute for Agri-food Technology, University of Lincoln, Lincoln, LN6 7TS UK

**Keywords:** Field studies, Nutrient concentration, Plant biomass, Potato cultivar, Yield

## Abstract

The application of arbuscular mycorrhizal (AM) fungi has been reported to confer multiple agronomic benefits to crop plants including cereals, vegetables, and fruit trees, as well as to improve soil structure and health. In this study, we conducted a meta-analysis to investigate whether AM fungal addition enhances potato yield. We further examined whether several experimental conditions (type of experiment, inoculation method, and source of AM fungi) and potato cultivar may explain the outcomes. We calculated the effect sizes of seven plant parameters by including a total of 106 independent pot and field experimental studies from 37 peer reviewed publications. Our results show that the addition of AM fungi has an overall positive effect on all potato plant parameters included in our analyses except for aboveground plant biomass. Potato cultivar was the main significant moderator explaining our findings, with some cultivars benefiting more from AM fungal presence than others. Our findings agree with several other global meta-analyses reporting positive effects of AM fungi on other important crops and highlights the potential application of these fungal symbionts in potato agro-ecosystems.

**Supplementary Information:**

The online version contains supplementary material available at 10.1007/s00572-024-01178-0.

## Introduction

One of the most imperative current environmental challenges is the sustainable production of food resources to feed the expanding global human population while reducing the amount of greenhouse gases present in the atmosphere and improving soil health (Wu et al. 2018). The need to limit global warming to 1.5 ºC is further highlighted in the latest IPCC report (2022): “Continued emission of greenhouse gases will cause further warming and long-lasting changes in all components of the climate system, increasing the likelihood of severe, pervasive, and irreversible impacts for people and ecosystems” (IPCC 2022, pp. 128). Therefore, to secure food production and for agriculture to be sustainable, we must integrate environmental aspects with technological improvements and economic profitability (Foley et al. 2011).

In this context, potato (*Solanum tuberosum* L.) has been identified as a key crop for global food security for several reasons. Potato is the most important non-cereal crop worldwide in terms of production (with an estimated 20 million hectares of farmland globally, Devaux et al. 2021) and human consumption (FAO 2021). Potato has a wide distribution range and offers high environmental adaptability (Haverkort et al. 2014). It has a high yield capacity (Devaux et al. 2020) and high macro- and micronutrient contents (Bailey et al. 2015). Potato is an important component of diversified cropping systems, has an increasing consumer demand, and has a long history of helping relieve food insecurities (Devaux et al. 2021). However, potatoes are one of the most dependent crops on chemical pesticides (Birch et al. 2012) and fertilisers (Wu et al. 2013). Potato plants are attacked by a wide range of organisms, including viruses, bacteria, fungi, Oomycetes, nematodes, and insects. Without crop protection, Oerke and Dehne (2004) estimated that about 71% of the attainable potato production would be lost to pests. On the other hand, potato is one of the most sensitive crops to soil water availability (both drought and excess) due to its relatively shallow root system (with most roots in the upper 30 cm of the soil profile, King et al. (2020) and the sparsity of the root hairs, which makes the plants’ root system rather inefficient at water and nutrient uptake (Yamaguchi 2002). Furthermore, potato production often relies on intensive cultivations and soil movement for seedbed preparation and harvest, with potentially negative consequences for soil biological systems (Hills et al. 2020).

One option to improve potato yield and quality while increasing soil health and decreasing the need for fertilisers and pesticides is the application of arbuscular mycorrhizal (AM) fungi. AM fungi are ubiquitous soil organisms that form a symbiotic relationship with the roots of most terrestrial plant species, including potatoes (e.g., Douds et al. 2007). In this type of symbiosis, the plant gives carbon (i.e., sugars and lipids) to the obligate biotrophic fungus in exchange for nutrients and water derived from the soil (Smith and Read 2008). The interaction usually translates into larger plants, with enhanced nutrient and water uptake compared to non-mycorrhizal plants, conferring better tolerance to both abiotic (Latef et al. 2016) and biotic (Dowarah et al. 2022) stresses.

AM fungi are naturally present in agricultural soils (Oehl et al. 2017), and AM fungi are commonly thought to play an important role for agroecosystem sustainability (Verbruggen et al. 2013; but see Ryan and Graham 2002, 2018). The benefits associated with AM fungi may be critical to increasing crop yields and productivity while mitigating the attendant soil and environmental footprint often associated with high input-intensive production systems (Vosatka and Dodd 2002). However, whether current cropping systems support AM fungi functioning remains unclear, fuelling discussions surrounding the relevance of AM fungi for agricultural production (Rillig et al. 2019; Brito et al. 2021). Most crops, including potatoes, naturally become colonised by AM fungi (Rúa et al. 2016). Studies in potatoes have shown that the addition of AM fungi can translate into increased tuber yield (Douds et al. 2007) and nutritional quality (Carrara et al. 2023), increasing water and nutrient uptake, and conferring plant tolerance against stresses (Liu et al. 2018). However, the evidence is mixed and mostly originates from experiments in controlled conditions, in which plants are cultivated in sterilised soil or substrates and inoculated with selected AM fungal species. This results in potential interactions between introduced AM fungi and resident soil fungi remaining largely unexplored. Given that AM fungi communities are modulated by the local environmental conditions and management practices (Jansa et al. 2006) and that the benefits of AM fungi addition to plant growth may be cultivar-dependent (Boussageon et al. 2023) and modulated by environmental conditions such as soil fertility (e.g., Bennett and Groten 2022), it becomes evident that the contribution of AM fungi to potato growth, yield, and/or nutrition may be context-dependent. Hence, to evaluate whether the addition of AM fungi improves potato crop yield and nutrition generally, we employed a meta-analytical approach to answer the following questions:

1. Does AM fungi addition improve potato tuber number and yield, plant root mass, shoot mass and P and N concentrations?
2. Which moderators influence the variation in plant responses to AM fungal inoculation?
3. Does the type of study (pot vs. field) influence the outcome and/or the magnitude of these effects?
4. Does publication bias (i.e., failure to report negative or statistically non-significant results) exist in the literature?

## Materials and methods

### Literature search and data extraction

We conducted an exhaustive literature search using Web of Knowledge (https://www.webofscience.com/wos/woscc/basic-search) on 1st November 2023 with the search terms [potato* AND mycorrhiza*] which returned 352 hits. After screening the titles and abstracts, studies were included if they fulfilled the following criteria:

1. The study reported addition of AM fungi and provided a control (i.e., no addition);
2. The study measured at least one of the following: reproductive yield (tuber number or mass), shoot mass, root mass, vegetative plant mass, plant P, N, or K concentration;
3. The mean, sample sizes, and standard deviation (SD), or standard error (SE) were reported or could be extracted from the study.

We also recorded metadata on the experimental conditions (field vs. non-field experiment), inoculation method (single taxon or multiple taxa), source of the AM fungal inoculum (commercial vs. not), and plant cultivar used to evaluate them as moderators if provided. Non-field experiments (called “pot experiments” hereafter) included mainly traditional pot experiments using sterilised substrate (autoclaved or heat-sterilised soil) with the addition of AM fungal inoculum. Only 3 pot experiments added AM fungal inoculum to unsterilised soil from the field. Few non-field experiments employed sterilised media different from soil.

In total, 36 peer reviewed papers were included which reported 106 independent studies. We considered independent studies within a paper those conducted for example on different field locations, studies done with different potato varieties, or different AM fungal inocula (see Supplementary Information Fig. S1 for the PRISMA workflow diagram detailing the meta-analysis process and List S1 for the list of papers included). When means and measures of variance were presented only in graphical form, these were extracted using WebPlotDigitizer (Rohatgi 2022). Standard deviations were back calculated from standard errors and sample sizes as SD = SE√n.

## Statistical analyses

All statistical analyses were carried out with R v.4.1.2 (R Core Team 2022). We computed Hedges’ g using the standardised mean difference with heteroscedastic population variances (SMDH) to estimate effect size (Bonett 2008, 2009). We employed the function ‘escalc’ in the package ‘metafor’ (Viechtbauer 2010). Effect sizes were calculated by subtracting the difference between plants inoculated with AM fungi minus control (i.e. non-inoculated) treatment plants. It should be noted that for field experiments, a non-inoculated treatment reflects only the lack of addition of AM fungi to the soil and thus, one could expect the presence of native mycorrhizal fungi even though in most studies that was not quantified. Effect sizes were weighted to ensure that robust studies (with small variances) were given added weight in the calculations (Harrison 2011). The weighted effect size was calculated using the inverse variance as the weight and is presented in the figures. Positive effect sizes indicate that the addition of AM fungi enhanced that particular trait compared to the control group (i.e., no AM fungi addition); whereas negative effect sizes indicate the opposite. Heterogeneity was assessed by inspection of forest plots and by calculating Cochran’s Q (Thompson and Sharp 1999) and I^2^ (Higgins and Thompson 2002; Higgins et al. 2003). Q is calculated as the weighted sum of squared differences between individual study effects and the pooled effect across studies and can be converted to I^2^, a statistic that describes the percentage of variation across studies that is due to heterogeneity rather than chance with the formula: I^2^ = 100(Q-df)/Q. Significant heterogeneity indicates that the studies do not share a common effect size.

Subsequently, to determine whether heterogeneity in effect sizes was explained by the four moderators considered (experiment type: field or pot, source of the AM fungal inoculum used: obtained commercially or not, AM fungal addition type: single species or cocktail, and plant cultivar), these factors were used as moderators in separate linear mixed-effects models using the “rma.mv” function from the metafor package (Viechtbauer 2010). The models were fed calculated effect sizes and sampling variances as described above and fitted using restricted maximum likelihood (REML). As we had multiple effect size values per study (e.g. different plots or years), we used a mixed model with Study ID as a random factor to consider the dependencies among estimates obtained from the same study.

Publication bias was visually inspected with funnel plots (i.e., a simple scatter plot showing the observed effect size on the ordinate axis against the standard error of the observed effect size on the abscissa). In the absence of publication bias and heterogeneity, one would expect to see the points forming a funnel shape, hence the name of the plot. We used a Rank correlation test to examine whether there was a significant asymmetry in the funnel plot, which may be indicative of publication bias (Begg and Mazumdar 1994).

## Results

### Overview

Following the PRISMA guidelines (Page et al. 2020), 36 peer reviewed research articles finally were selected for meta-analysis. The number of observations for each parameter investigated are shown in Table 1. Half of the observations in our meta-analysis were obtained from pot experiments (54/106). Most studies employed the addition of single AM fungal species (80/106) and among these, *Rhizophagus irregularis* was the most abundant AM fungal species used (57/80). A similar number of observations were made using commercially available inoculum versus not (51 vs. 54, respectively). Forty-four different potato cultivars were tested, with Fripapa being used the most (18/106). Publication bias was detected for four of the seven parameters included in this meta-analysis (Table 1, Fig. S2).

**Table 1:** Summary of the results of the meta-analysis investigating whether the addition of AM fungi significantly affects several potato plant parameters. Effect size (Hedge’s g) was calculated as the standardised mean difference with heteroscedastic population variances (SMDH). Heterogeneity is expressed as the Q estimate, followed by I^2^. Statistical significance is indicated with asterisks (p values: < 0.001 = ***, 0.001–0.01 = **, 0.01–0.05 = *)

Parameter	Included studies	Effect size (Hedges’ g)	Heterogeneity (Q and I^2^)	Publication bias (Kendall’s tau)
Tuber number	55	0.80 ± 0.23 ***	115.23 *** 53%	0.2375 *
Tuber yield	84	1.37 ± 0.30 ***	650.5 *** 87%	0.2826 ***
Aboveground mass	35	0.33 ± 0.30	111.51 *** 70%	-0.0572
Root mass	32	0.99 ± 0.29 **	135.92 *** 77%	0.2661 *
Total plant mass	27	1.48 ± 0.50 **	121.33 *** 79%	0.2877 *
Plant P concentration	25	1.13 ± 0.38 **	430.51 *** 92%	0.1500
Plant N concentration	22	1.33 ± 0.30 ***	121.72 *** 92%	0.1273

## Tuber number and yield

**Fig. 1:**
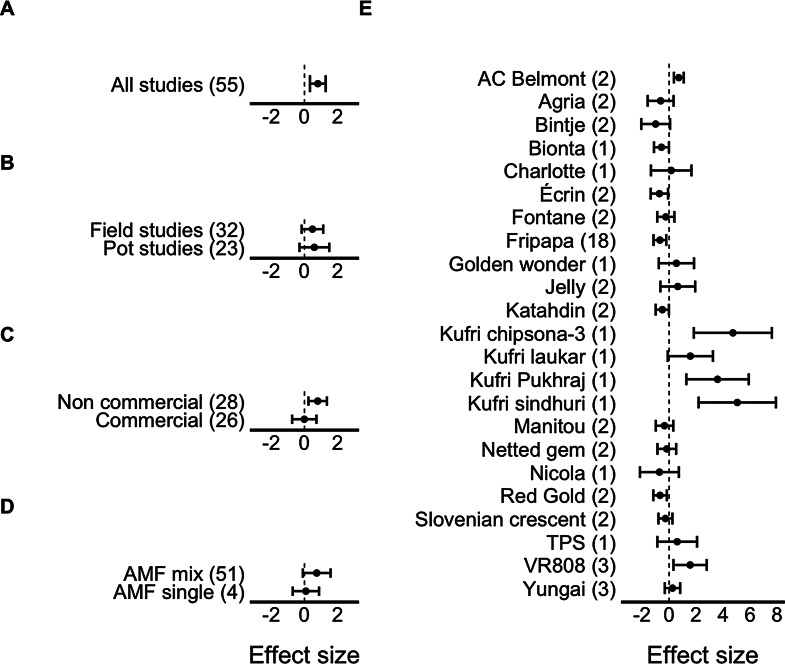
Effect sizes (model estimates, mean ± 95% CI) for the total number of tubers produced including (**A**) all studies, (**B**) for field vs. pot studies, (**C**) non-commercial vs. commercial AMF inocula, (**D**) AM fungal mix vs. single species inocula, and (**E**) for each potato cultivar. Dotted line shows Hedge’s g = 0. When the confidence interval does not include zero, the effect size is statistically significant. Numbers in parentheses represent the number of cases included

The number of tubers produced per plant ranged between 2.0 and 92.2 (9.0 ± 1.6, mean ± SE) and between 1.8 and 151.4 (9.0 ± 2.6) in plants with and without AM fungi inoculation, respectively, with a significant positive estimated effect size, indicating that the addition of AM fungi had a positive effect on the number of tubers produced (Table 1; Fig. 1A). There was significant heterogeneity among the studies included (Table 1), suggesting that other experimental treatments or moderators may have influenced the results. Whether the experiment was conducted in pots or in the field (Q_1_ = 1.68, *p* = 0.19), and the type (Q_1_ = 0.04, *p* = 0.83) and source of AM fungal inoculum (Q_1_ = 0.00, *p* = 0.98) did not affect the effect size (Fig. 1B-D). Potato cultivar was the only significant moderator (Q_22_ = 84.44, *p* < 0.01). Out of the 23 different potato cultivars used, the addition of AM fungi had a significant effect on 10 of them: for studies performed with AC Belmont, Kufri Chipsona-3, Kufri Pukhraj, Kufri Sindhuri, and VR808 cultivars, AM fungal addition increased the total number of tubers produced, but for Bionta, Écrin, Fripapa, Katahdin, and Red Gold cultivars, AM fungal addition had a negative effect on tuber number (Fig. 1E).

**Fig. 2:**
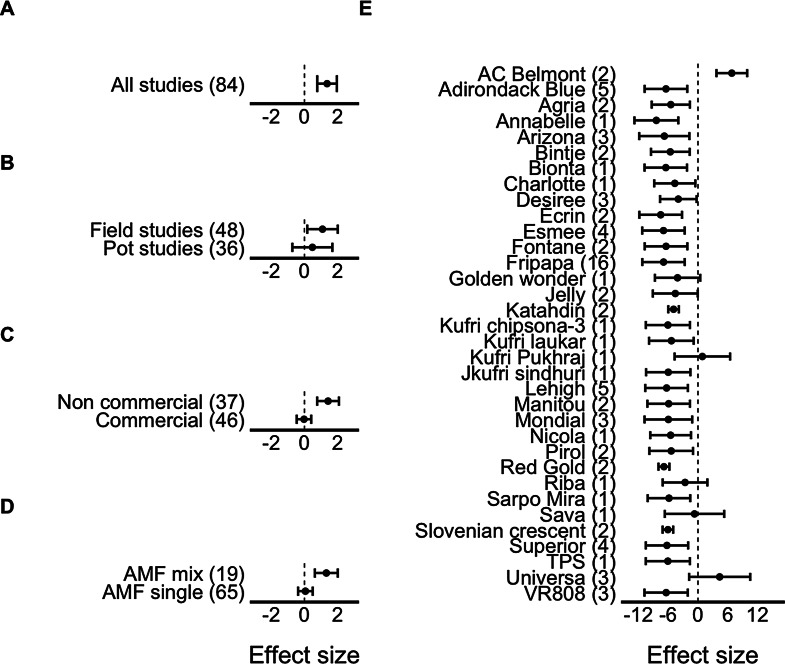
Effect sizes (model estimates, mean ± 95% CI) for the tuber yield including (**A**) all studies, (**B**) for field vs. pot studies, (**C**) non-commercial vs. commercial AM fungal inocula, (**D**) AM fungal mix vs. single species inocula, and (**E**) for each potato cultivar. Dotted line shows Hedge’s g = 0. When the confidence interval does not include zero, the effect size is statistically significant. Numbers in parentheses represent the number of cases included

Similarly, tuber yield per plant ranged between 1.1 and 16,400 g (mean 928.1 ± 241.1) and between 1.2 and 12,510 g (mean 859.4 ± 220.1) in plants with and without AM fungi inoculation, respectively, with a significant estimated effect size of 1.37 ± 0.30, indicating that the addition of AM fungi had also a positive significant effect on the tuber yield (Fig. 2A). Heterogeneity was significant (Table 1), suggesting moderators may have affected the results. In this case, potato cultivar was again the only significant moderator (Q_33_ = 232.2, *p* < 0.01; Fig. 2E). For this variable, among the 34 different potato cultivars used, AM fungal addition had a significant negative effect on tuber yield on most cultivars except six. For Golden wonder, Kufri pukhraj, Riba, Sava, and Universa, AM fungal addition did not affect the effect size for tuber yield, but for AC Belmont, the effect was positive (Fig. 2E).

### Plant mass and nutrient concentrations

Data on plant aboveground dry mass were available from 35 studies, with a mean ± SE of 7.6 ± 1.8 g (range = 0.61–42.30 g) and 7.0 ± 1.6 g (range = 0.78–37.20 g) with and without AM fungi inoculation, respectively, and the addition of AM fungi did not have a significant effect (Table 1; Fig. S3). Heterogeneity was significant (Table 1) but none of the tabulated moderators significantly explained the heterogeneity in the data (Fig. S3).

**Fig. 3:**
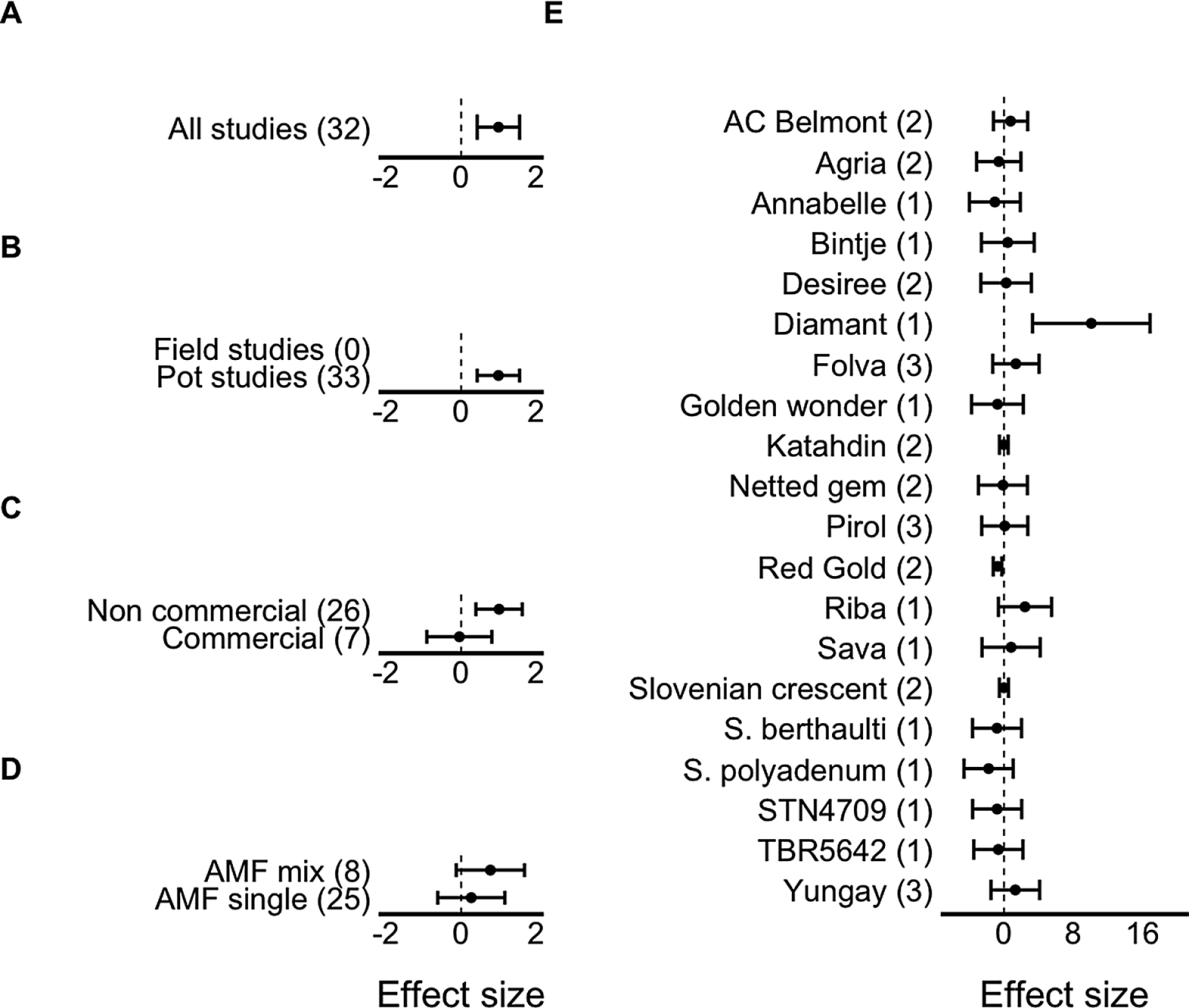
Effect sizes (model estimates, mean ± 95% CI) for root mass including (**A**) all studies, (**B**) for field vs. pot studies, (**C**) non-commercial vs. commercial AM fungal inocula, (**D**) AM fungal mix vs. single species inocula, and (**E**) for each potato cultivar. Dotted line shows Hedge’s g = 0. When the confidence interval does not include zero, the effect size is statistically significant. Numbers in parentheses represent the number of cases included

This result was different for root biomass (range: 0.2–5.2 g and 0.1–3.6 g for AM and non-inoculated plants, respectively), with a significant positive estimated effect size due to AM fungal inoculation (Table 1; Fig. 3). For these parameters, both the type of experiment (Q_31_ = 135.92, *p* < 0.01) and the potato cultivar used (Q_19_ = 40.23, *p* = 0.003) were significant moderators, even though all included cases were pot studies (Fig. 3B). AM fungal addition had a significant positive effect on root mass for Diamant cultivar but a negative one for Red Gold (Fig. 3E). Finally, for total plant dry mass (range: 0.004–117.7 g and 0.004–76.1 g for AM and non-inoculated plants, respectively), the effect of AM fungi addition was significantly positive (Table 1; Fig. S4). For this parameter, type of experiment (Q_1_ = 4.00, *p* = 0.04) and cultivar (Q_16_ = 100.6, *p* < 0.01) again were significant moderators (Fig. S3), as positive effects of AM fungi addition were observed in field studies, but negative effects were reported for pot studies. Regarding cultivars, AM fungi were beneficial for five cultivars, with Sava benefiting the most, and negative for 3 cultivars (Fig. S4).

Regarding plant nutrient concentrations, AM fungal addition did significantly increase P and N concentrations in potato plants (Table 1; Fig. S5 and S6). P concentration (mg/g DW leaf) ranged between 0.8 and 525.0 in AM plants and between 0.8 and 106.0 in non-inoculated plants. In both cases, potato cultivar was the only significant moderator. For P concentration, the effect was positive for 11 cultivars and negative for the cultivar AC Belmont, whereas for N concentration (range: 11.2–41.4 mg/g and 11.2–41.2 mg/g in AM and non-inoculated plants), Lehigh cultivar benefitted the most (Table 1; Fig. S5 and S6).

## Differences among cultivars

A detailed analysis of the differences among the 44 cultivars reported indicates contrasting effects of the addition of AM fungi depending on whether yield parameters, plant size or nutrient concentrations are considered (Fig. S7). The addition of AM fungi increased tuber number in 5 cultivars, but a decrease was observed in another 5 cultivars and no significant effect was observed in 13 cultivars (Fig. S7). Tuber yield was the parameter that benefitted the least from AM fungi, as AM fungal addition had a negative effect in all reported but six cultivars. Plant aboveground biomass was largely unaffected in most cultivars. Conversely, the effect of AM fungal addition on total plant biomass was positive for 4 cultivars but negative for another 4. For P concentrations, most cultivars had a positive response (Fig. S7). Overall, the cultivar AC Belmont appeared to be the one that benefitted the most broadly from AM fungi addition both in terms of number of tubers produced and overall yield.

## Discussion

The present meta-analysis investigated whether the introduction of AM fungi improves potato plant parameters. We observed that overall, the addition of AM fungi significantly increased all plant parameters considered except for plant aboveground mass. Our findings agree with several meta-analysis reporting positive effects of AM fungi on other crops such as wheat (Pellegrino et al. 2015) and other food cereal crops (Zhang et al. 2019; Neidhardt 2020). Moreover, our results suggest that whether studies were performed in the field or in pots did not influence the results, but highlight the importance of considering plant potato cultivar, as this was an important significant moderator explaining our results.

### Does AM fungal addition improve potato plant parameters?

The beneficial effects of AM fungal inoculation on plant performance (both plant biomass and reproductive output) are widely established, and thus we expected to find positive effects also in potatoes. We found significant effect sizes of AM fungal introduction for all plant parameters considered but plant aboveground mass. The effects of AM fungi on plant resource allocation towards roots and shoots are quite complex and may not always translate to an increase in plant reproductive structures (Zhang et al. 2015). A meta-analysis by Veresoglou et al. (2012) analysed the allometric partition of plant biomass into shoots and roots and concluded that AM fungal colonisation resulted in reallocation of biomass towards shoots (and away from roots). That effect depended, however, on whether plants were exposed to stress. Moreover, results from a meta-analysis by Qin et al. (2022) in which the effects of AM fungi on plants were compared among 5 plant families indicated that AM fungi had a lower effect size on plant biomass in the Solanaceae than the other families, even though the mechanisms were not discussed. Crops from this family usually are grown in agricultural lands that are heavily fertilised and thus, this could reduce the reliance on AM fungi for nutrient acquisition. Perhaps in agreement with that, we did find that the presence of AM fungi had a positive effect size on root biomass. As far as we are aware, predicting plant responsiveness to AM fungi is complex, with plant phylogeny and plant traits only partially explaining the variation observed (Reinhart et al. 2012). Full explanation of plant responsiveness to AM fungi likely requires consideration of the evolution of both AM plant phylogenetic lineages and of AM fungal genera (Hoeksema et al. 2018).

Our meta-analysis indicates that the effect of AM fungal addition generally translates into the well-established for other plant species increased P and N concentrations also in potato plants, which may translate into an increase in plant mass. Regardless of the effects on plant mass and nutrient concentrations, the most important effects for potato growers likely are the increase in the total number of tubers produced and their size. Our study confirms the results of Hijiri (2016), who demonstrated the feasibility of AM fungal inoculation in field conditions using 231 field trials that employed the same AM fungal inoculant over a 4-year period and at least 31 different potato cultivars. In their study, AM fungal addition increased the total marketable yield of potato from 38.3 ± 1.03 tons/ha to 42.2 ± 1.03 tons/ha (unfortunately, this study did not include a measure of SD/SE, so we could not include these data in our meta-analysis) With these promising results in mind, it is perhaps surprising to see that AM fungal addition is not widely practiced. Ryan and Graham (2002) and Ryan et al. (2019) reviewed the literature on the role of AM fungi in commercial agroecosystems and concluded that these plant symbionts are not important. The reasons given were diverse and included the relatively expensive production costs associated with the mass production of AM fungal inoculum (see e.g., Benami et al. 2020), alongside the relatively high soil nutrient contents and frequent soil disturbances in agrosystems, which might hinder AM fungal applicability. In addition, the use of fungicides might also be a major deterrent to the functioning and natural nutrient uptake capacity of AM fungi (Edlinger et al. 2022) and the potential unintended ecological consequences of introducing bioinoculants in natural systems should also be considered (Hart et al. 2018; Basiru and Hijiri 2022). Therefore, addition of AM fungal inoculum in the field often translates into little impact in the agricultural sector (Tarbell and Koske 2007), even though the results of our meta-analysis indicate that AM fungi can be beneficial in potato agrosystems, in line with a similar study on tomato, another solanaceous crop (Bona et al. 2017).

### Which moderators influence the variation in potato plant response to AM fungal inoculation?

Potato cultivar was a significant moderator of most parameters analysed, and the type of experiment was a significant moderator for plant root and total biomass. Differences between cultivars may arise from the differences in mycorrhizal dependency/responsiveness due to domestication (see e.g., Chu et al. 2013; Mao et al. 2014), as different cultivars may possess different symbiosis traits (i.e., inherited traits that underlie host responses to beneficial microbes and/or regulate their colonisation, see Porter and Sachs 2020; but see Sawers et al. 2010 and Galván et al. 2011 for a comprehensive discussion of the topic). Because current plant cultivars are the result of domestication of wild species through cultivation practices and artificial selection, some authors recently have suggested that domestication has contributed to the disruption of plant-microbe symbioses (Porter and Sachs 2020), as breeding and crop improvement may have resulted in the loss of certain genes, for example by selecting for resistance to fungal pathogens (Smith and Goodman 1999). Our findings suggest that domestication in potato only partially can explain the results, because among the 45 cultivars included in our meta-analysis, AC Belmont seems to be the one that benefitted the most from AM fungal inoculation, even though it is neither the newest nor oldest cultivar. However, more than 4800 potato varieties exist, highlighting continued investment in breeding programs (Naeem et al. 2021).

A key question is whether AM fungal species diversity translates into functional diversity and thus into improved crop yield, or whether one or two key species are needed (Chagnon et al. 2021). Our analysis indicates that there were no differences between single AM fungal species or a mix, but most of the observations in our meta-analysis came from single AM fungal additions, and most of those used *Rhizophagus irregularis*. Clearly this highlights the need to include more AM fungal species and more combinations (but see Ryan and Graham 2018).

### Does the type of study (pot vs. field) influence the outcome and/or the magnitude of these effects?

Extrapolating findings from pot studies to field studies in AM fungal research is usually deemed as challenging partially because of the simplification involved in pot systems. In addition to the identity of the host and the fungi involved (e.g., Hoeksema et al. 2018), several abiotic factors such as soil pH, nutrient concentration and even the experiment duration (e.g., Qin et al. 2022) are known to influence AM fungal activity and the responses of the hosts to the usually mutualistic symbionts. However, our analysis indicates that the type of study considered (i.e., whether conducted in pots or in the field) mostly gave similar effect sizes for most of the variables considered (but note that for plant N concentration only field studies were available, and for root biomass only pot studies were available). The most striking difference between pots and field studies was detected in plant total mass, where overall, pot studies gave a significant negative effect size, but a positive and significant one for field studies. Several potential explanations might explain this difference, including that on average, pots studies were performed using soils of overall higher fertility than field studies (P concentration mean ± SD: 1393.8 ± 2386.6 versus 54.7 ± 40.0 mg/Kg; N concentration mean ± SD: 5.98 ± 4.00 versus 0.76 ± 0.89 in pot vs. field studies, respectively).

### Does publication bias exist in the literature?

Our analyses confirm that publication bias exists among studies reporting AM fungal effects on potato plants. Publication bias is a well-established fact regarding the scientific literature, and our analyses indicate that there was positive outcome bias (i.e., statistically significant effects or studies reporting positive AM fungal inoculation results seem to be more likely to be published than those reporting negative or neutral results) in studies reporting AM fungal effects on potato systems. To prevent publication bias, the scientific community should adopt transparent reporting practices such as the pre-registration of studies and the publication of results regardless of their outcomes (i.e., replication studies, negative results and null findings).

## Conclusions

To summarise, our results indicate that the addition of AM fungi has an overall positive effect on potato plant parameters, including yield. Potato cultivar was the main overall significant moderator. It is well established that the interaction between AM fungi and their plant hosts is complex and context dependant. Other moderators such as soil pH, soil nutrient concentrations, or even the experimental duration could influence the effect of AM fungi on the potato plant parameters measured. Although these factors are predicted as highly influential drivers of AM fungal activity, the selected studies did not all consistently report such variables and thus could not be included. For example, only 15 papers reported soil P concentration, and from these, only 7 specified the extractant used. Changes in such variables may have an impact on the development and activity of AM fungi, which may then influence the overall growth and performance of host plants. Nevertheless, our study suggests that application of AM fungi enhances plant productivity in potato crops.

## Electronic supplementary material

Below is the link to the electronic supplementary material.

Supplementary Material 1

## Data Availability

The data will be uploaded into FigShare upon publication.
