# Supplementary material for: Is arbuscular mycorrhizal fungal addition beneficial to potato systems? A meta-analysis
Source: Mycorrhiza. 2024 Dec 16;35(1):5. doi: 10.1007/s00572-024-01178-0 (PMC11649713; doi:10.1007/s00572-024-01178-0)
Supplement: Supplementary file 1 — Supplementary Material 1 [file 572_2024_1178_MOESM1_ESM.docx]

**Is arbuscular mycorrhizal fungal addition beneficial to potato systems? A meta-analysis**

Segun Oladele^1,2,3^, Iain Gould^2,4^, Sandra Varga^1,5*^

^1^School of Natural Sciences, University of Lincoln, Lincoln LN6 7TS, UK

^2^Lincoln Institute for Agri-food Technology, University of Lincoln, Lincoln LN6 7TS, UK

*Corresponding author: [svarga@lincoln.ac.uk](mailto:svarga@lincoln.ac.uk) or [sandravarga30@hotmail.com](mailto:sandravarga30@hotmail.com)

^3^https://orcid.org/0000-0002-7589-8658

^4^ https://orcid.org/0000-0001-5315-2241

^5^https://orcid.org/0000-0001-9799-714X

**Figure S1**. Preferred Reporting Items for Systematic Reviews and Meta-Analysis (PRISMA) flow describing the screening and decision process and the complete number of studies included in the meta-analysis.


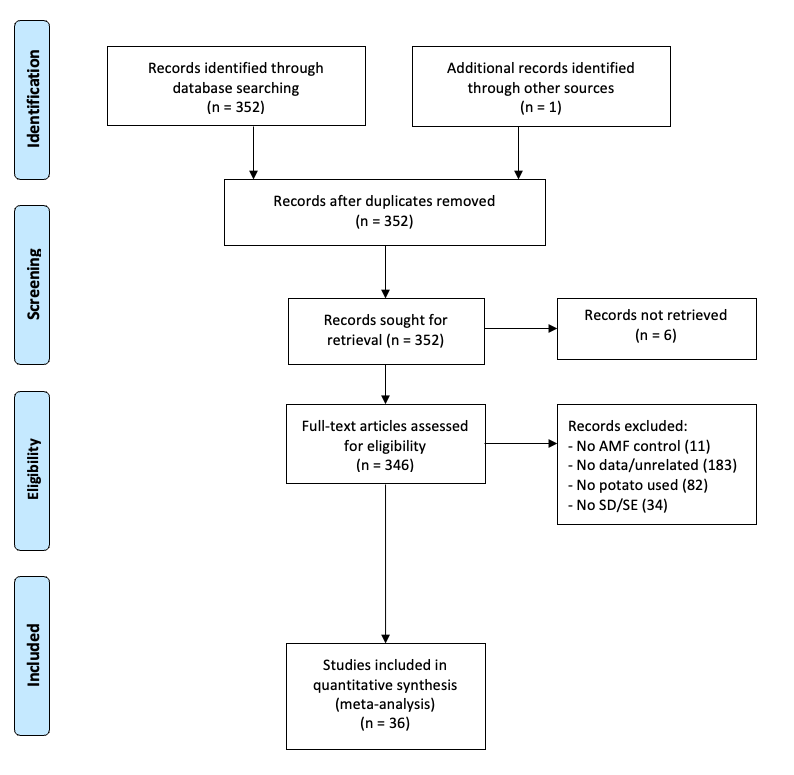


**List S1.** List of studies included in the meta-analysis.

Alarcon S, Tereucan G, Cornejo P, Contreras B, Ruiz A (2022) Metabolic and antioxidant effects of inoculation with arbuscular mycorrhizal fungi in crops of flesh-coloured *Solanum tuberosum* treated with fungicides. Journal of the Science of Food and Agriculture 102: 2270-2280.

Alaux PL, Cesar V, Naveau F, Cranenbrouck S, Declerck S (2018) Impact of *Rhizophagus irregularis* MUCL 41833 on disease symptoms caused by *Phytophthora infestans* in potato grown under field conditions. Crop Protection 107: 26-33.

Bagy HMMK, Hassan EA, Nafady NA, Dawood MFA (2019) Efficacy of arbuscular mycorrhizal fungi and endophytic strain *Epicoccum nigrum* ASU11 as biocontrol agents against blackleg disease of potato caused by bacterial strain *Pectobacterium carotovora* subsp. *atrosepticum* PHY7. Biological Control 134: 103-113.

Barogh RB, Hassanpanah D, Esmaeilpour B, Godehkahriz SJ, Jari SK (2023) Co-inoculation of arbuscular mycorrhizal fungi and plant growth-promoting Rhizobacteria improve growth, biochemical attributes, and nutritional status of potato (*Solanum tuberosum* L.) minitubers. Journal of Soil Science and Plant Nutrition 23: 3447-3460.

Bell CA, Magkourilou E, Barker H, Barker A, Urwin PE, Field KJ (2023) Arbuscular mycorrhizal fungal-induced tolerance is determined by fungal identity and pathogen density. Plants People Planet 5: 241-253.

Bell CA, Magkourilou E, Field KJ, Urwin PE (2023) Sequence of introduction determines the success of contrasting root symbionts and their host. Applied Soil Ecology 182: 104733.

Bell CA, Magkourilou E, Urwin PE, Field KJ (2022) Disruption of carbon for nutrient exchange between potato and arbuscular mycorrhizal fungi enhanced cyst nematode fitness and host pest tolerance. New Phytologist 234: 269-279.

Bennett AE, Millar NS, Gedrovics E, Karley AJ (2016) Plant and insect microbial symbionts alter the outcome of plant-herbivore-parasitoid interactions: implications for invaded, agricultural and natural systems. Journal of Ecology 104: 1734-1744.

Boussageon R, van Tuinen D, Lapadatescu C, TrTpanier M, Vermersch E, Wipf D, Courty PE (2023) Effects of field inoculation of potato tubers with the arbuscular mycorrhizal fungus *Rhizophagus irregularis* DAOM 197,198 are cultivar dependent. Symbiosis 89: 213-226.

Buysens C, Alaux PL, Cesar V, Huret S, Declerck, S, Cranenbrouck S (2017) Tracing native and inoculated *Rhizophagus irregularis* in three potato cultivars (Charlotte, Nicola and Bintje) grown under field conditions. Applied Soil Ecology 115: 1-9.

Buysens C, Cesar V, Ferrais F, de Boulois HD, Declerck S (2016) Inoculation of Medicago sativa cover crop with *Rhizophagus irregularis* and *Trichoderma harzianum* increases the yield of subsequently-grown potato under low nutrient conditions. Applied Soil Ecology 105: 137-143.

Carrara JE, Reddivari L, Lehotay SJ, Zinati G, Heller WP (2023) Arbuscular mycorrhizal fungi increase the yield and nutritional quality of yellow and purple fleshed potatoes (*Solanum tuberosum*). American Journal of Potato Research 100: 210-220.

Davies FT, Calderon CM, Huaman, Z (2005) Influence of arbuscular mycorrhizae indigenous to Peru and a flavonoid on growth, yield, and leaf elemental concentration of 'Yungay' potatoes. Hortscience 40: 381-385.

Deja-Sikora E, Werner K, Hrynkiewicz, K (2023) AMF species do matter: *Rhizophagus irregularis* and *Funneliformis mosseae* affect healthy and PVY-infected *Solanum tuberosum* L. in a different way. Frontiers in Microbiology 14: 1127278.

Douds DD, Nagahashi G, Reider C, Hepperly PR (2007) Inoculation with arbuscular mycorrhizal fungi increases the yield of potatoes in a high P soil. Biological Agriculture & Horticulture 25: 67-78.

Gabriel-Neumann E, Neumann G, Leggewie G, George E (2011) Constitutive overexpression of the sucrose transporter SoSUT1 in potato plants increases arbuscular mycorrhiza fungal root colonization under high, but not under low, soil phosphorus availability. Journal of Plant Physiology 168: 911-919.

Ghobadi M, Dehnavi MM, Yadavi AR, Parvizi K, Zafari D (2020) Reduced P fertilization improves Fe and Zn uptake in potato when inoculated with AMF in P, Fe and Zn deficient soil. Rhizosphere 15: 100239.

Graham SO, Green NE, Hendrix JW (1976) Influence of vesicular-arbuscular mycorrhizal fungi on growth and tuberization of potatoes. Mycologia 68: 925-929.

Hills K, Collins H, Yorgei G, McGuire A, Kruger C (2020) Improving soil health in Pacific Northwest potato production: a review. American Journal of Potato Research 97: 1-22.

Ismail Y, Hijri M (2012) Arbuscular mycorrhisation with *Glomus irregulare* induces expression of potato PR homologues genes in response to infection by *Fusarium sambucinum*. Functional Plant Biology 39: 236-245.

Lalaymia I, Naveau F, Arias AA, Ongena M, Picaud T, Declerck S, Calonne-Salmon M (2022) Screening and efficacy evaluation of antagonistic fungi against *Phytophthora infestans* and combination with arbuscular mycorrhizal fungi for biocontrol of late blight in potato. Frontiers in Agronomy 4: 948309.

Liu C, Liu F, Ravnskov S, Rubaek GH, Sun Z, Andersen MN (2017) Impact of wood biochar and its interactions with mycorrhizal fungi, phosphorus fertilization and irrigation strategies on potato growth. Journal of Agronomy and Crop Science 203: 131-145.

Liu CX, Liu SRF, Liu FL, Rubaek GH, Andersen MN (2018) Arbuscular mycorrhizal fungi alleviate abiotic stresses in potato plants caused by low phosphorus and deficit irrigation/partial root-zone drying. Journal of Agricultural Science 156: 46-58.

Loit K, Soonvald L, Adamson K, Runno-Paurson E, Tedersoo L, Astover A (2023) Assessing the effect of commercial arbuscular mycorrhizal fungal inoculum on potato plant disease incidence, yield and the indigenous root fungal community composition. Biocontrol 68: 537-547.

Lojan P, Senes-Guerrero C, Suarez JP, Kromann P, Schussler A, Declerck S (2017) Potato field-inoculation in Ecuador with *Rhizophagus irregularis*: no impact on growth performance and associated arbuscular mycorrhizal fungal communities. Symbiosis 73: 45-56.

Lombardo S, Abbate C, Pandino G, Parisi B, Scavo A, Mauromicale G (2020) Productive and physiological response of organic potato grown under highly calcareous soils to fertilization and mycorrhization management. Agronomy 10: 1200.

Lone R, Alaklabi A, Malik JA, Koul KK (2020) Mycorrhizal influence on storage metabolites and mineral nutrition in seed propagated potato (*Solanum tuberosum* L.) plant. Journal of Plant Nutrition 43: 2164-2175.

Louche-Tessandier D, Samson G, Hernandez-Sebastia C, Chagvardieff P, Desjardins Y (1999) Importance of light and CO2 on the effects of endomycorrhizal colonization on growth and photosynthesis of potato plantlets (*Solanum tuberosum*) in an in vitro tripartite system. New Phytologist 142: 539-550.

Pathak D, Lone R, Khan S, Koul KK (2019) Isolation, screening and molecular characterization of free-living bacteria of potato (*Solanum tuberosum* L.) and their interplay impact on growth and production of potato plant under mycorrhizal association. Scientia Horticulturae 252: 388-397.

Rodriguez-Morelos VH, Declerck S, Calonne-Salmon M (2023) Azoxystrobin alters the dynamics of short-term phosphorus uptake of mycorrhizal potato plants associated to *Rhizophagus irregularis*. Journal of Plant Nutrition and Soil Science 186: 95-104.

Ryan NA, Deliopoulos T, Jones P, Haydock PPJ (2003) Effects of a mixed-isolate mycorrhizal inoculum on the potato - potato cyst nematode interaction. Annals of Applied Biology 143: 111-119.

Saini I, Kaushik P, Al-Huqail AA, Khan F, Siddiqui, MH (2021) Effect of the diverse combinations of useful microbes and chemical fertilizers on important traits of potato. Saudi Journal of Biological Sciences 28: 2641-2648.

Terry V, Kokkoris V, Villeneuve-Laroche M, Turcu B, Chapman K, Cornell C, Zheng ZM, Stefani F, Corradi, N (2023) Mycorrhizal response of *Solanum tuberosum* to homokaryotic versus dikaryotic arbuscular mycorrhizal fungi. Mycorrhiza 33: 333-344.

Thiem D, Szmidt-Jaworska A, Baum C, Muders K, Niedojadło K, Hrynkiewicz K. (2014) Interactive physiological response of potato (*Solanum tuberosum* L.) plants to fungal colonization and Potato virus Y (PVY) infection. Acta Mycologica 49: 291-303.

Velivelli SLS, Kromann P, Lojan P, Rojas M, Franco J, Suarez JP, Prestwich, BD (2015) Identification of mVOCs from Andean rhizobacteria and field evaluation of bacterial and mycorrhizal inoculants on growth of potato in its center of origin. Microbial Ecology 69: 652-667.

Yang Q, Ravnskov S, Andersen MN (2020) Nutrient uptake and growth of potato: Arbuscular mycorrhiza symbiosis interacts with quality and quantity of amended biochars. Journal of Plant Nutrition and Soil Science 183: 220-232.

Yang Q, Ravnskov S, Pullens JWM, Andersen MN (2022) Interactions between biochar, arbuscular mycorrhizal fungi and photosynthetic processes in potato (*Solanum tuberosum* L.). Science of the Total Environment 816: 151649.

**Figure S2**. Funnel plots for the different plant variables included in the meta-analysis. The X axis shows the effect size (standardised mean difference) and Y-axis show the inverse standard error of the effect size as an index of precision.


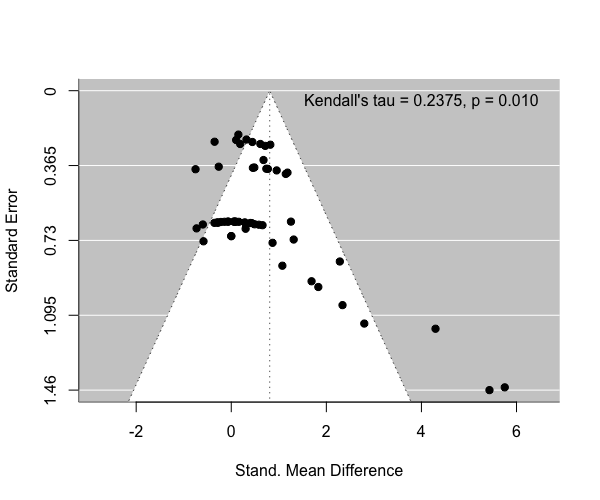

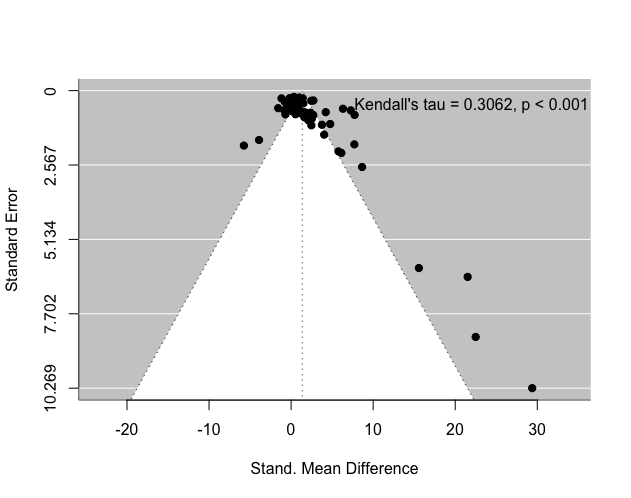

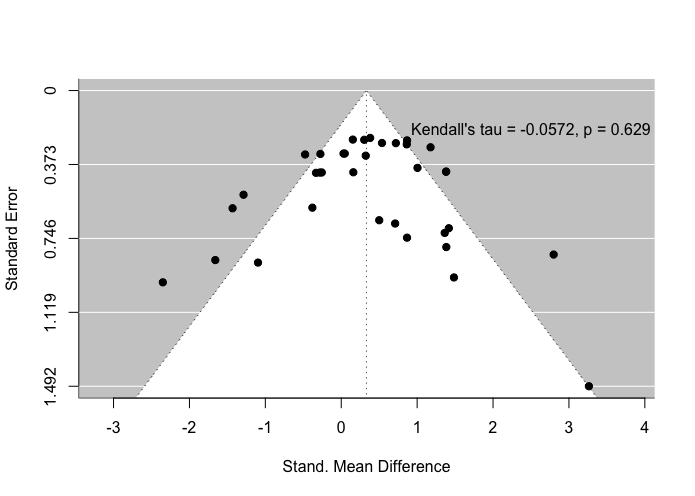

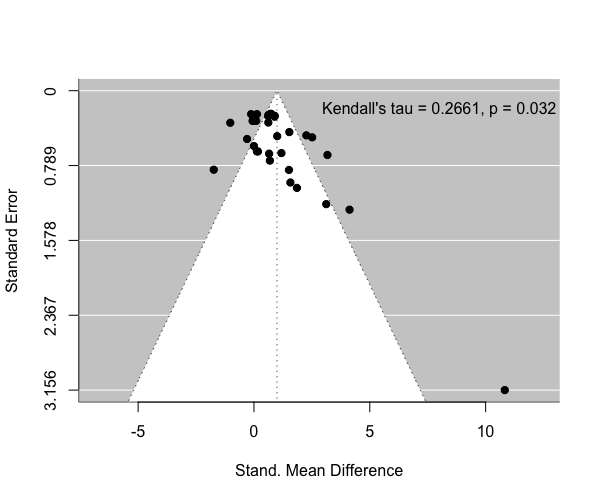


**B**. Tuber yield.

**A**. Tuber number.

**D**. Root mass.

**C**. Aboveground plant mass.


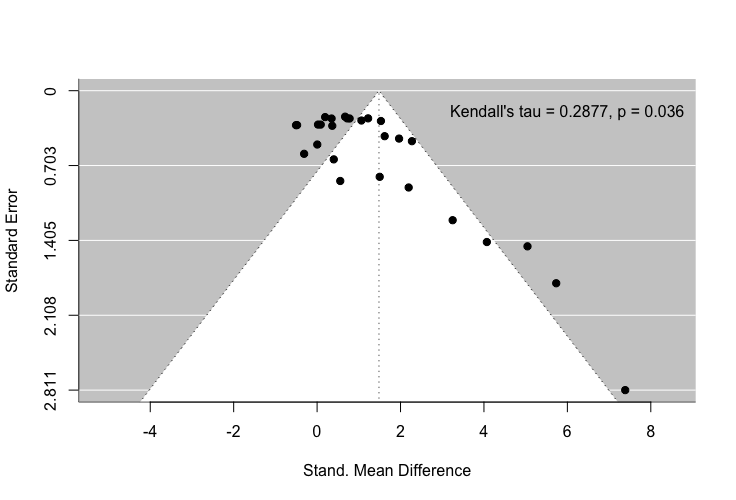

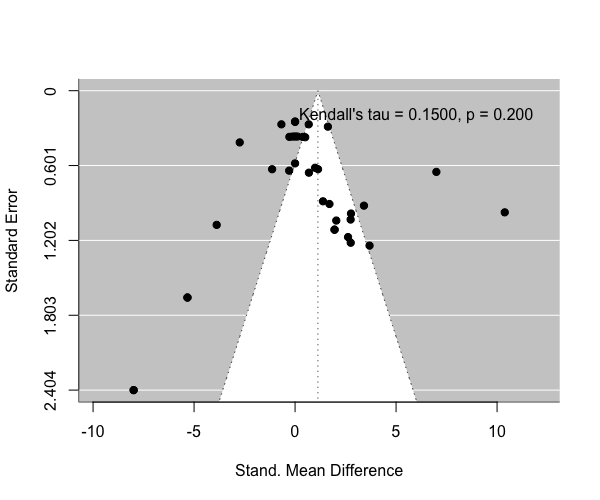

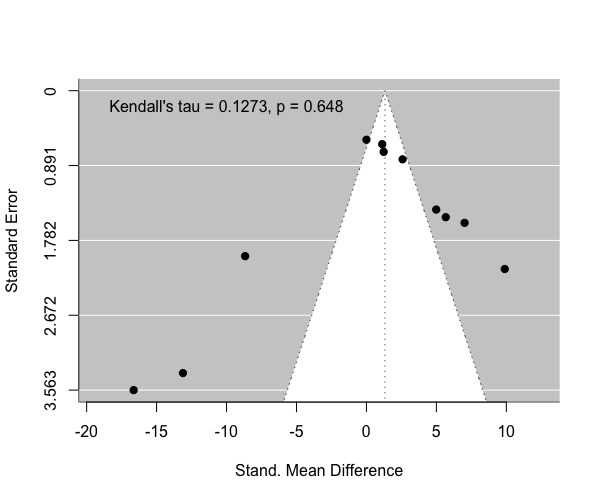


**F**. Plant P content.

**E**. Total plant mass.

**G**. Plant N content.

**Fig. S3**. Effect sizes (model estimates, mean ± 95% CI) for plant aboveground dry mass including (A) all studies, (B) for field vs. pot studies, (C) non-commercial vs commercial AM fungal inocula, (D) AM fungal mix vs. single species inocula, and (E) for each potato cultivar. Dotted line shows Hedge’s g = 0. When the confidence interval does not include zero, the effect size is statistically significant. Numbers in brackets represent the number of cases included.


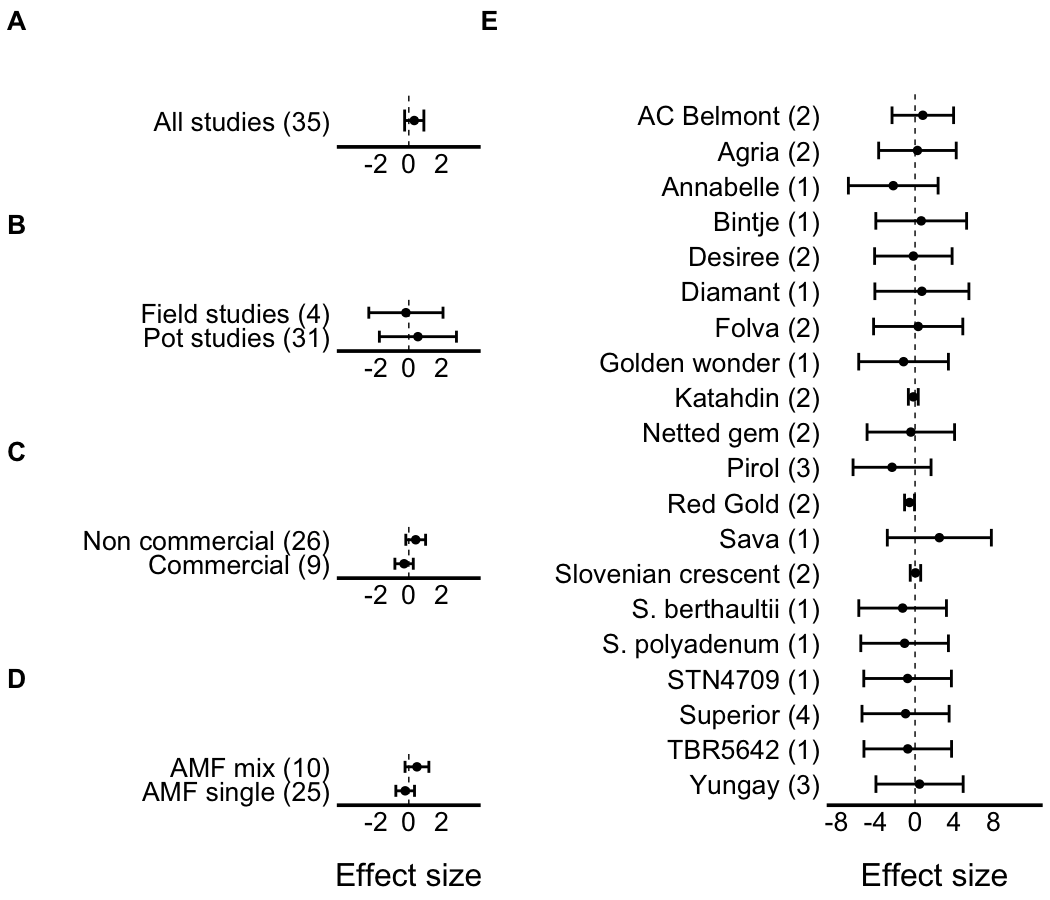


**Fig. S4**. Effect sizes (model estimates, mean ± 95% CI) for the total plant biomass including (A) all studies, (B) for field vs. pot studies, (C) non-commercial vs commercial AM fungal inocula, (D) AM fungal mix vs. single species inocula, and (E) for each potato cultivar. Dotted line shows Hedge’s g = 0. When the confidence interval does not include zero, the effect size is statistically significant. Numbers in brackets represent the number of cases included.

**
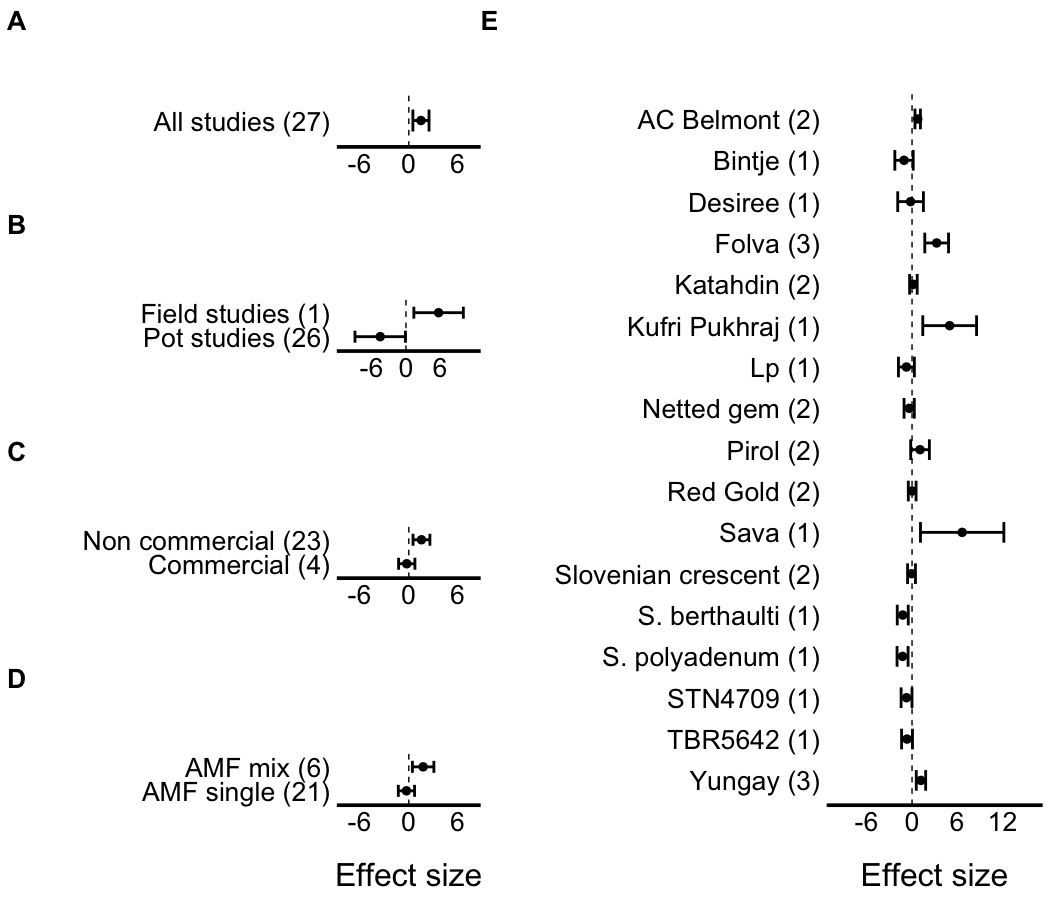
**

**Fig. S5**. Effect sizes (model estimates, mean ± 95% CI) for the plant P content including (A) all studies, (B) for field vs. pot studies, (C) non-commercial vs commercial AM fungal inocula, (D) AM fungal mix vs. single species inocula, and (E) for each potato cultivar. Dotted line shows Hedge’s g = 0. When the confidence interval does not include zero, the effect size is statistically significant. Numbers in brackets represent the number of cases included.


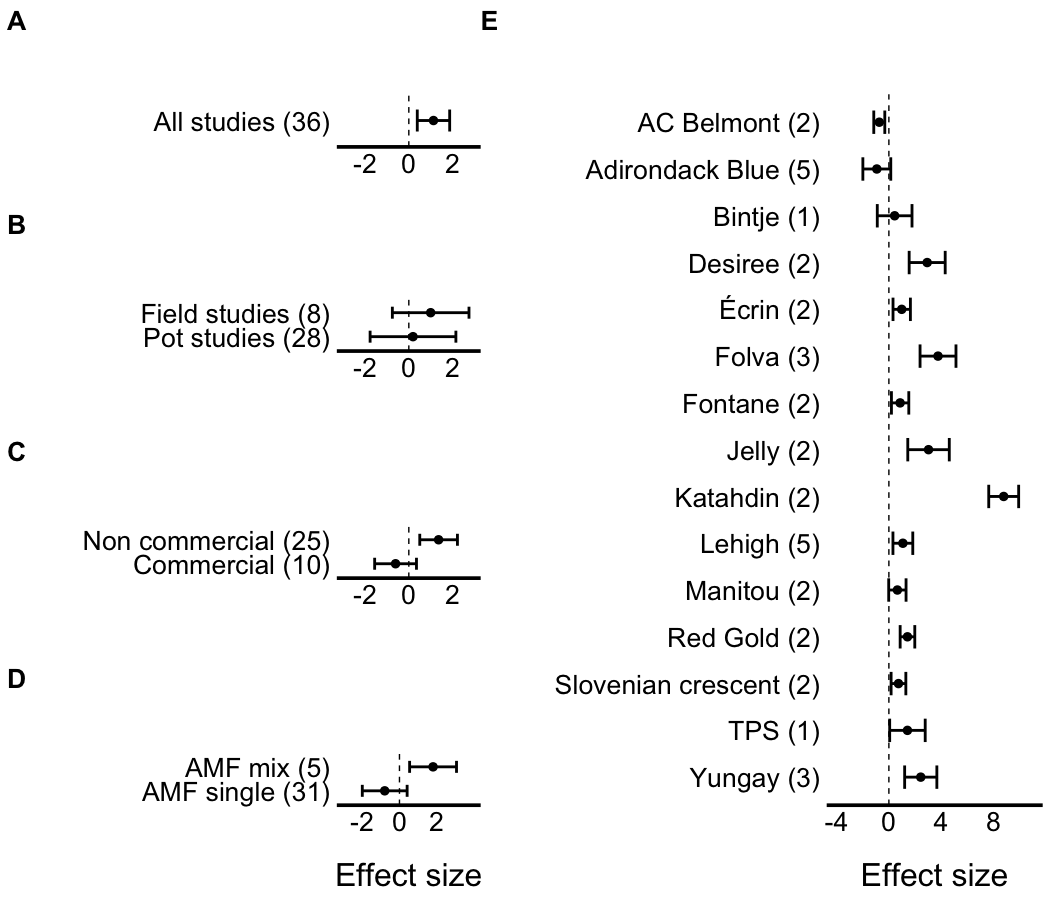


**Fig. S6**. Effect sizes (model estimates, mean ± 95% CI) for the plant N content including (A) all studies, (B) for field vs. pot studies, (C) non-commercial vs commercial AM fungal inocula, (D) AM fungal mix vs. single species inocula, and (E) for each potato cultivar. Dotted line shows Hedge’s g = 0. When the confidence interval does not include zero, the effect size is statistically significant. Numbers in brackets represent the number of cases included.


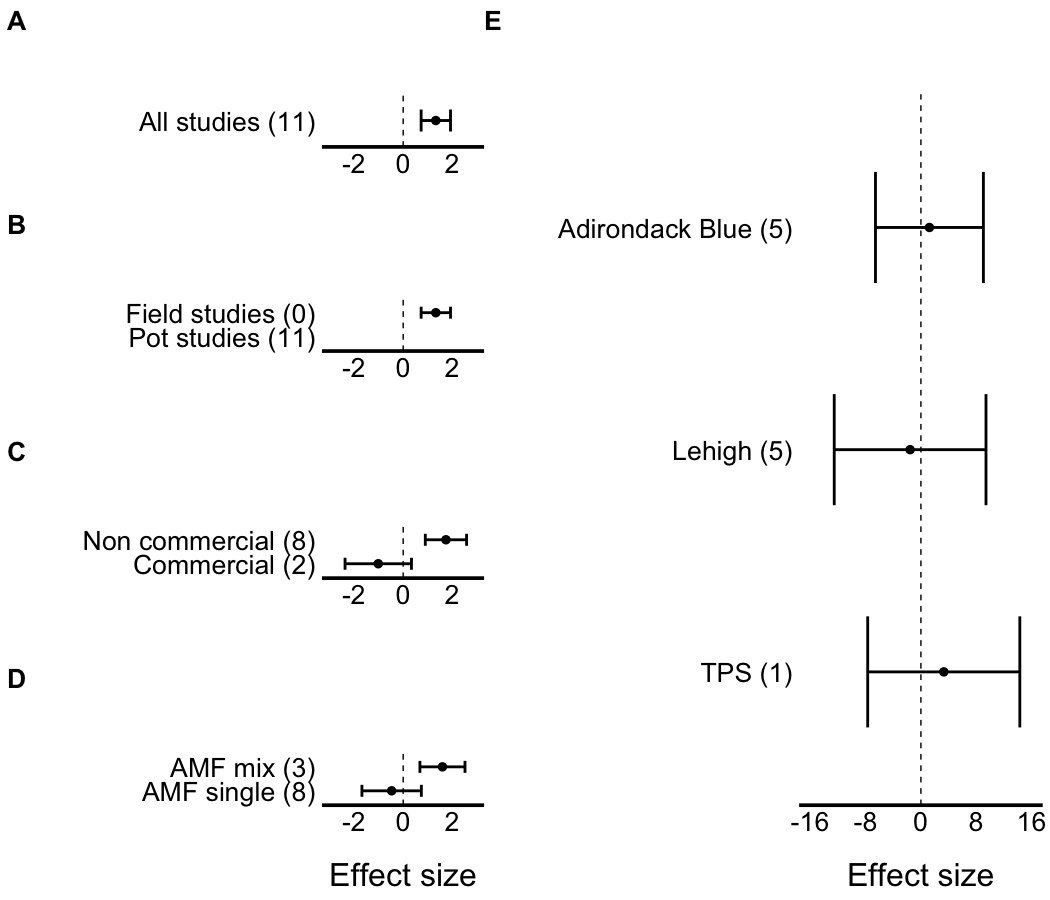


**Fig. S7.** Heat map showing whether addition of AM fungi translated to a statistically significant positive (green boxes) or negative (red boxes) effects on the different potato cultivars found in the meta-analysis. Non statistically significant results are indicated with grey boxes.
